# Irisin: A New Code Uncover the Relationship of Skeletal Muscle and Cardiovascular Health During Exercise

**DOI:** 10.3389/fphys.2021.620608

**Published:** 2021-02-01

**Authors:** Chunlian Ma, Haichao Ding, Yuting Deng, Hua Liu, Xiaoling Xiong, Yi Yang

**Affiliations:** ^1^College of Health Science, Wuhan Sports University, Wuhan, China; ^2^Graduate School, Wuhan Sports University, Wuhan, China

**Keywords:** irisin, myokine, exercise, skeletal muscle, cardiovascular health

## Abstract

Exercise not only produces beneficial effects on muscle itself *via* various molecular pathways, but also mediates the interaction between muscles and other organs in an autocrine/paracrine manner through myokines, which plays a positive role in maintaining overall health. Irisin, an exercise-derived myokine, has been found involved in the regulation of some cardiovascular diseases. However, the relationship between irisin and cardiovascular health is not fully elucidated and there are some divergences on the regulation of irisin by exercise. In this review, we present the current knowledge on the origin and physiology of irisin, describe the regulation of irisin by acute and chronic exercises, and discuss the divergences of the related research results. Importantly, we discuss the role of irisin as a biomarker in the diagnosis of cardiovascular diseases and describe its treatment and molecular mechanism in some cardiovascular diseases. It is expected that irisin will be used as a therapeutic agent to combat cardiovascular diseases or other disorders caused by inactivity in the near future.

## Introduction

Cardiovascular disease is the most common underlying cause of death, accounting for 31.5% of all deaths worldwide. It is estimated that 43.9% of the adult population in the United States will suffer from some form of cardiovascular disease by 2030, and the total global medical costs of this disease will reach $918 billion (Benjamin et al., 2017). Although progress in clinical treatment and care has reduced the mortality rate of patients with cardiovascular disease, the incidence of the disease continues to increase, and good prevention and treatment strategies are still needed. It is well-established that physical exercise reduces all-cause mortality and increases longevity (Moore et al., 2012). Particularly, exercise reduces the risk of cardiovascular diseases, regulates the abnormal metabolism of blood lipid, and improves vascular function (Diabetes, 2012). Exercise plays an important role in the prevention and treatment of hypertension and coronary heart disease, which has become an effective management in maintaining overall health (Fiuza-Luces et al., 2018).

As the largest endocrine organ, the skeletal muscle secretes a hormone known as myokine. Irisin is a myokine secreted by the skeletal muscle both in rodents and in humans (Bostrom et al., 2012; Lourenco et al., 2019), which enters into circulation during or immediately after physical activity (Cooper et al., 2018). Owing to properties of driving white adipose tissue browning (Wu and Spiegelman, 2014), alleviating insulin resistance (Bostrom et al., 2012; Yuksel Ozgor et al., 2020), improving glucose homeostasis, and liver lipid accumulation (Zhang et al., 2013; Kurdiova et al., 2014), irisin is emerging as a key molecular for metabolic diseases and other disorders known to improve with exercise (Seo et al., 2020). An increasing number of studies show that the concentrations of circulating irisin in patients with some kinds of cardiovascular disease has changed compared with normal people (Shen et al., 2017; Wang et al., 2017; Hisamatsu et al., 2018). Especially, it has been demonstrated that the application of irisin can affect the pathological processes and improve the disease state of certain cardiovascular diseases (Li et al., 2018; Fan et al., 2020; Yin et al., 2020).

In this review, we summarize the origin of irisin and discuss the regulation of irisin by exercise. Equally, we focus on the key role of irisin in the diagnosis of cardiovascular diseases, as well as the therapeutic effects and molecular mechanisms of certain cardiovascular diseases. We hope to at least partially bridge the knowledge gap between skeletal muscle and cardiovascular health during exercise and provide new ideas for the prevention and treatment of cardiovascular diseases.

## Secretion of Irisin From Skeletal Muscle

Irisin is a hormone composed of 112 amino acids, which was first discovered in 2012 by Bostrom et al., and named after the Greek messenger goddess Iris (Bostrom et al., 2012). In their original work, the muscle of transgenic mice overexpressing peroxisome proliferator-activated receptor-γ coactivator 1α (PGC1α) stimulates an increase in the synthesis of transmembrane fibronectin type III domain-containing protein 5 (FNDC5), which is cleaved and secretes irisin (Bostrom et al., 2012). Following the initial study, it is soon reported that the start codon of human FNDC5 gene is atypical ATA rather than ATG of rodents, and the translation efficiency of human FNDC5 gene constructed with ATA start codon is found to be very low in HEK293 cells (Raschke et al., 2013). Erickson et al. considered that Bostrom’s experiment had serious flaws, and FNDC5 may be just a transmembrane receptor (Erickson, 2013). The claim that human FNDC5 gene codes for irisin has been questioned (Albrecht et al., 2015), and some scholars believe that the beneficial effects of irisin observed in mice are unlikely to appear in humans (Raschke et al., 2013; Elsen et al., 2014). However, there are indeed cases of proteins being expressed from unusual start codons of genes in humans, such as VANGL2, FGFR1, KCNN4, and TRPV6 (Ivanov et al., 2011). Is irisin also expressed from the unusual start codon in human? To address the issue, Jedrychowski et al. used improved mass spectrometry technique with synthetic peptides rich in heavy stable isotopes (six 13c atoms) as the internal standards and found that irisin is mainly expressed from the non-canonical start codon of FNDC5 (Jedrychowski et al., 2015; Polyzos et al., 2015). The *in vitro* experiments conducted by Raschke et al. (2013) may not reflect the real situation *in vivo*. The level of circulating irisin is higher than insulin and lower than leptin, with the molecular weight of 12 kDa (Jedrychowski et al., 2015). While other studies have reported that the molecular weight of irisin is 15–32 kDa due to glycosylation or incomplete deglycosylation (Bostrom et al., 2012; Lee et al., 2014; Zhang et al., 2014). Recently, it was illustrated that irisin reversed intestinal epithelial barrier dysfunction after intestinal ischemia reperfusion injury *via* binding to integrin αVβ5 (Bi et al., 2020). Similarly, Kim and colleagues have shown that irisin binds to proteins of αV class of integrins, and further biophysical studies identify interacting surfaces between irisin and αVβ5 integrin. Additionally, chemical inhibition of the αV integrins blocks the signaling pathway activated by irisin both in osteocytes and fat cells (Kim et al., 2019). These studies suggest that the membrane receptors of irisin exist as some scholars have predicted, and the αVβ5 integrin is the receptor of irisin in osteocytes, adipocytes, and enterocytes (Greenhill, 2019; Kim et al., 2019; Bi et al., 2020). Even so, whether there are irisin receptors in other cells, such as cardiomyocytes or vascular endothelial cells, needs further study. Anyhow, the discovery of irisin receptors above mentioned has opened a window for scholars to conduct in-depth studies.

**Figure 1:**
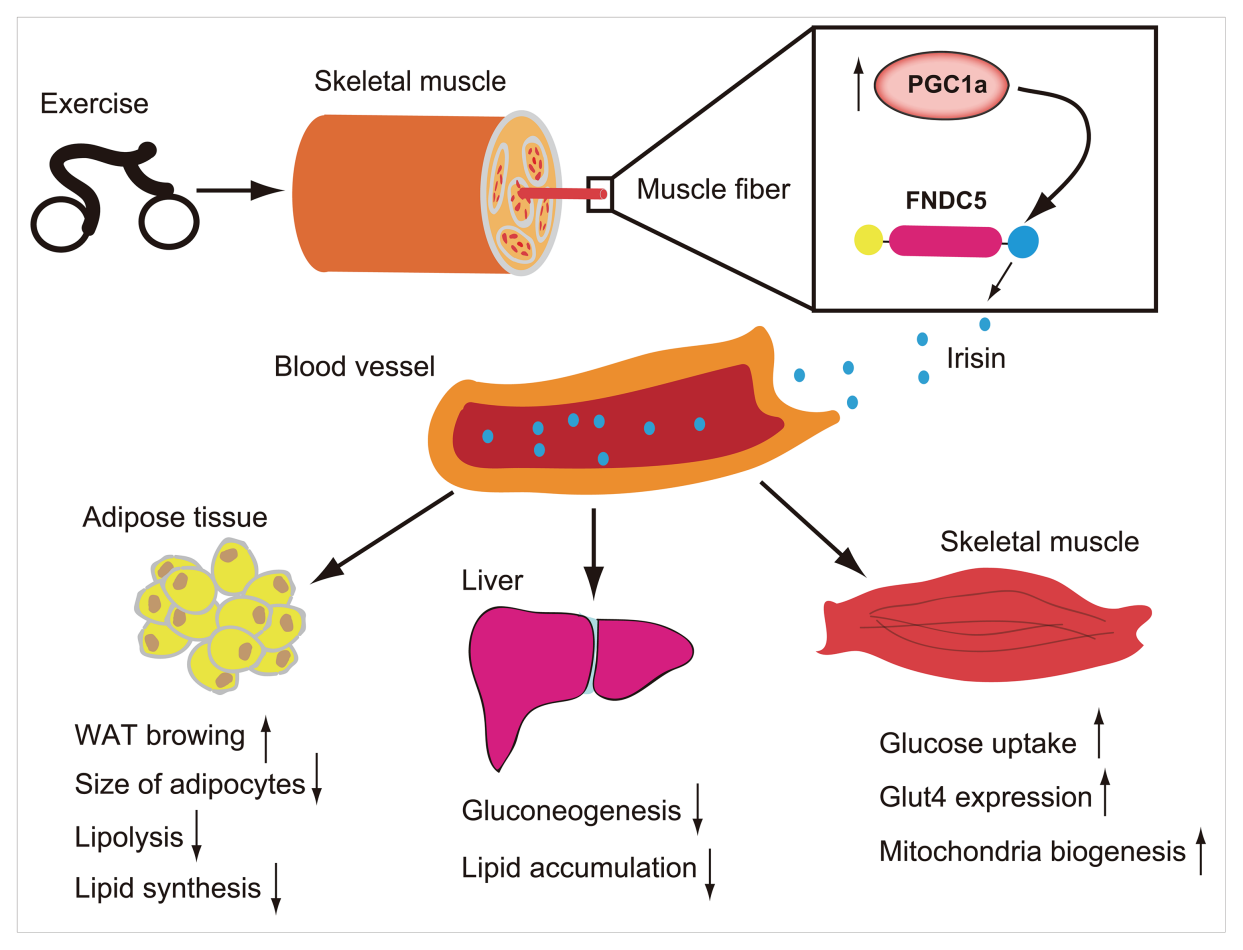
Exercise induces increase of PCG1a expression in skeletal muscle, which in turn drives the production of membrane protein fibronectin type III domain-containing protein 5 (FNDC5). The FNDC5 is cleaved and secretes irisin (blue ball). Irisin enters in the blood circulation and participates in the metabolic regulation of lipid and glucose in some organs such as skeletal muscle, liver, and adipose tissue.

In humans, FNDC5 mRNA is mainly expressed in muscles and other muscle containing organs, such as pericardium, rectum, and, artery (Huh et al., 2012), and can be detected in the blood, saliva, cerebrospinal fluid, and bronchoalveolar lavage fluid (Aydin et al., 2013; Sanchis-Gomar et al., 2014; Shen et al., 2017; Ruan et al., 2018). Muscle mass is the main predictor of higher circulating irisin levels in humans (Huh et al., 2012), and age-related muscle mass loss may lead to lower circulating irisin levels in elderly people (Tu et al., 2018). Nevertheless, it was also demonstrated that circulating irisin levels increased with the increase of fat mass, particularly in obesity (Perakakis et al., 2017) and correspondingly decreased with the decrease of fat mass after bariatric surgery (Huh et al., 2012). Given the irisin expression in the muscle is 200-fold of that in adipocytes (Moreno-Navarrete et al., 2013), and its key roles in lipid metabolism, it is possible that there is an irisin compensation mechanism, particular in the obese. A large number of studies have shown that irisin has a potential role in some metabolic diseases such as diabetes, obesity, and participates in the regulation of energy metabolism. For example, it promotes browning of white adipose tissue (Wu and Spiegelman, 2014), increases thermogenesis (Bostrom et al., 2012), reduces lipid accumulation, and maintains glucose homeostasis in skeletal muscle, liver, and other organs (Kurdiova et al., 2014; Perakakis et al., 2017; Figure 1). Abnormal glucose and lipid metabolism, diabetes, and obesity are risk factors of cardiovascular diseases. As an important regulator of energy metabolism, irisin has a potential role in maintaining cardiovascular health.

## Regulation of Irisin by Exercise

### Acute Exercise Increases Circulating Irisin Concentration

As a myokine, irisin is produced by the contraction of skeletal muscle. According to the reports, circulating irisin concentrations increase significantly when the muscle ATP level decreases and remain unchanged as the muscle ATP level remains unchanged (Huh et al., 2012). ATP decline, a state of metabolic demand, may be the initial signal that stimulates irisin secretion to defend muscle ATP homeostasis during exercise (Huh et al., 2012; Bell et al., 2016). A series of studies have shown that acute exercise is a potential stimulus to promote the secretion of irisin (Huh et al., 2015; Fatouros, 2018; Fox et al., 2018). It has been reported that circulating irisin concentrations of young healthy adults increase significantly 30 min after an acute exercise (Huh et al., 2012). Furthermore, a single 40 min of aerobic running can induce minimal increase of serum irisin in both hot (21~25°C) and cold (−5~5°C) environment (Ozbay et al., 2020). Data from animal studies have found that the concentrations of serum irisin increase significantly in hyperthyroidism and hypothyroidism rats after forced swimming with 5% of the weight-bearing body weight for 100 min (Samy et al., 2015). Additionally, one time of high-intensity interval exercise (HIIE), moderate-intensity exercise (CME), or resistance exercise (RE) can significantly increase the circulating irisin concentrations both in healthy people and in patients with metabolic syndrome detected by ELISA kit (Huh et al., 2015). A meta-analysis also shows that the concentrations of circulating irisin in adults increase significantly immediately after an acute bout of exercise, and fitness level is an important factor affecting the effect (Fox et al., 2018).

As everyone knows, resistance training can improve muscle mass more effective than endurance training. Accordingly, it was reported that resistance training promoted irisin secretion more than endurance training or endurance and resistance combined training (Tsuchiya et al., 2015). This finding is consistent with other studies showing that the circulating irisin concentrations are higher after RE than that of HIIE or CME (Huh et al., 2015). On the contrary, it was reported recently that the circulating irisin concentrations in physically active young males did not change after immediate, 30 and 90 min of vigorous-intensity continuous training (VICT, 85%VO_2_max) compared with that before exercise (Reycraft et al., 2020). Interestingly, 30 min later, the irisin concentrations are significantly higher than that immediately after exercise (Reycraft et al., 2020). Although the results of this study are not exactly the same as the previous studies from Huh et al. as well as Samy et al. (Huh et al., 2015; Samy et al., 2015), it is believed that exercise at least promotes the secretion of irisin, which is reflected in the significant difference between immediately and 30 min after exercise, in spite of no significant difference in irisin concentrations before and after exercise. Exercise intensity should be an important factor affecting the secretion or the elimination of irisin. Because lower exercise intensity (65% VO_2max_) does not induce significant increase of circulating irisin concentrations 30 min after exercise compared to immediately after exercise, unlike higher intensity (85% VO_2max_) at the same study (Reycraft et al., 2020). This research is consistent with the study of Tsuchiya et al., showing that high-intensity exercise causes greater irisin response compared with low-intensity exercise under similar energy consumption (Tsuchiya et al., 2014).

Acute exercise protocols represent potent stimuli for irisin release if they are characterized by adequate intensity and/or duration (Fatouros, 2018). Of note, the maximum irisin concentration appears immediately after exercise and decreases 1 h later in one study (Huh et al., 2015), while other studies show that the maximum irisin concentration appears 30 min to 1 h after exercise (Tsuchiya et al., 2014; Reycraft et al., 2020). It suggests that time course changes of irisin concentration in response to acute exercise are complex. In addition to the factors such as exercise intensity, duration and subjects’ fitness level, a more optimized exercise program that includes a detailed time course is needed to explore the response of irisin to acute exercise in the future.

### Chronic Exercise Improves Irisin Metabolic Dynamic and Selectively Increases the Circulating Irisin Concentration

Chronic exercise produces multiple positive effects on overall health in many ways. As a myokine, irisin has received increasing attention in the research related to chronic exercise and overall health. Some studies have reported that chronic exercise increases circulating irisin concentration. For instance, the concentrations of plasma irisin in rodents increase significantly after 3 weeks of free wheel running (Bostrom et al., 2012). In humans, 10 weeks of moderate intensity cycling (65%VO_2_max), 20–35 min per day and 4–5 times per week, make a 2-fold rise of irisin concentrations, detected by plasma FNDC5 expression (Bostrom et al., 2012). Similarly, circulating FNDC5 levels in some young male athletes are several times higher than those in middle-aged obese women (Huh et al., 2012), which may be related to higher muscle mass *via* exercise training year after year in athletes. Moreover, the baseline plasma irisin concentration is 3.6 ng/ml in sedentary individuals and significantly increases to 4.3 ng/ml in individuals undergoing 12 weeks of high-intensity aerobic training (Jedrychowski et al., 2015). Furthermore, the circulating irisin concentrations of physically active subjects are higher than that of sedentary, and that of rural are higher than urban subjects (Moreno et al., 2015). This may be due to the fact that rural residents often engage in physical labor, which can exercise muscles similar to physical activity.

However, other studies are inconsistent with the results mentioned above, which has aroused widespread concern and controversy among scholars (Timmons et al., 2012; Hofmann et al., 2014; Spiegelman and Wrann, 2014; Fatouros, 2018). Hecksteden et al. have found that 26 weeks of moderate aerobic exercise (60% HRmax), including walking and running, 45 min per day and three times per week, or strength training with an intensity of 20 repetition maximum (RM), two times of 15 repetitions, three times per week, improves the physical performance of healthy subjects aged 30–60 years (Hecksteden et al., 2013). However, the detection of frozen serum by ELISA kit found that neither aerobic exercise nor strength training caused an increase in the concentration of circulating irisin (Hecksteden et al., 2013). Similarly, Huh also reported that after an 8-week repeated sprint training of moderately trained young male, the circulating irisin concentrations detected by ELISA kit remained unchanged compared to before training (Huh et al., 2012). Additionally, 6 weeks of whole body vibration exercise failed to increase the baseline circulating irisin concentration of healthy untrained females (Huh et al., 2014). Collectively, results from human and animal studies above-mentioned point to different directions, and it seems difficult to draw firm conclusions about the irisin response to chronic exercise.

Interestingly, although the baseline irisin concentrations remained unchanged after 6 weeks of chronic exercise, the circulating irisin levels increased from 9.5 to 18.1% immediately after an acute bout of vibration exercise (Huh et al., 2014). It suggests that chronic exercise promotes the metabolic dynamic and secretion efficiency of irisin. Furthermore, it has been found that 16–20 weeks of moderate intensity exercise (70% HRmax) does not increase circulating irisin concentrations of normal pigs, but increases the circulating irisin concentrations in hypercholesterolemic pigs (Fain et al., 2013). In addition, 6 weeks of intense endurance cycling promoted muscle FNDC5 messenger RNA (mRNA) expression by 30% in elderly subjects, which failed in young subjects (Timmons et al., 2012). It was also reported that the circulating irisin concentrations in older adults increased with the moderate intensity of cardiovascular training program, but not in young (Miyamoto-Mikami et al., 2015; Gmiat et al., 2017). This indicates that the regulation of irisin by chronic exercise may be population-selective. Obese people, the elderly, and patients with metabolic dysfunction may be more sensitive to chronic exercise. In fact, sample fresh or not, reliability of testing methods may be other factors that affect the research results and cause research divergences (Hecksteden et al., 2013; Huh et al., 2015; Albrecht et al., 2020). To fully understand the response of irisin to chronic exercise, the age, gender, health, or disease status of subjects need to be well-controlled in future studies.

## Irisin and Cardiovascular Health

### Irisin Could Be a Biomarker for Diagnosis of Cardiovascular Diseases

Excessive accumulation of fat, high cholesterol, and metabolism disorders are prone to cause cardiovascular diseases (Angosta et al., 2020). While circulating irisin concentrations are negatively correlated with risk factors of cardiovascular health, such as hyperglycaemia, triglycerides, visceral adiposity, and extramyocellular lipid deposition (Kurdiova et al., 2014). Moreover, it has been reported that serum irisin concentrations are inversely associated with the prevalence of coronary artery calcification (CAC) after adjusting for age and behavioral factors of Japanese men aged 40–79 years (Hisamatsu et al., 2018). Further, after adjustment for cardiometabolic risk factors, the inverse association between serum irisin concentration and CAC progression still persisted (Hisamatsu et al., 2018). This suggests that circulating irisin concentrations have the potential role to predict occurrence and development of CAC. However, another study has found that under a sedentary lifestyle, rather than active, circulating irisin concentrations are positively correlated with cardiovascular risk factors such as fasting insulin and fasting triglycerides (Moreno et al., 2015). It is possible that in the preclinical stage of some kinds of cardiovascular disease, there may be a compensatory increase in the circulating irisin levels under an inactive lifestyle. Additionally, muscle mass is an important factor affecting circulating irisin concentration (Huh et al., 2012; Sesti et al., 2014; Mai et al., 2020), and the difference in muscle mass among subjects may also be a reason for the divergence of research results. In future studies, it is worth trying to use the ratio of irisin concentration/muscle mass as a biomarker to predict disease and judge pathological processes.

Furthermore, multivariable logistic regression analysis reveals that serum irisin concentration is an independent determinant of the presence of coronary artery disease (CAD; Deng, 2016). Negative correlation between serum irisin concentrations and atherosclerosis index has been found, and the serum irisin concentrations in patients with CAD are significantly lower than that of healthy controls (Deng, 2016). It suggests that those with decreased serum irisin concentrations are more likely to develop coronary atherosclerosis. Similarly, it has been shown that different severity of CAD corresponds to different serum irisin levels in patients with stable angina, suggesting that serum irisin can be used to predict the severity of CAD (Efe et al., 2017). In addition, a meta-analysis based on 741 studies involving 876 patients with CAD and 700 controls, reported that circulating irisin concentration was 18.10 ng/ml lower in patients with CAD or atherosclerosis than those in healthy controls (Guo et al., 2020). Moreover, the serum irisin concentrations of Type-2 diabetic women are significantly lower than that of the normal controls, while the serum irisin concentrations of diabetic patients with atherosclerosis are lower than that of diabetic patients without atherosclerosis (Saadeldin et al., 2018). It indicates that circulating irisin has the potential implication as a diagnostic biomarker for monitoring the progression of cardiovascular disease in diabetic patients. Notably, the irisin concentrations in patients with chronic cardiovascular disease are stable (Panagiotou et al., 2014), whereas the irisin concentrations decrease gradually in saliva and serum within 48 h after acute myocardial infarction (AMI; Aydin et al., 2014). The results above indicate that irisin in body fluids has important clinical value to be used as a diagnostic indicator for the development of AMI.

### Effect and Mechanism of Irisin on Cardiovascular Diseases

#### Atherosclerosis

Atherosclerosis is a common type of cardiovascular disease, characterized by lipids accumulation on the wall, which may lead to arterial rupture and stenosis (Nguyen et al., 2019). It has been demonstrated that circulating irisin concentrations are negatively correlated with the parameters of atherosclerosis, such as coronary atherosclerosis index (CAI) and carotid intima-media thickness (cIMT; Deng, 2016; Icli et al., 2016). In addition, irisin concentrations are significantly lower in type 2 diabetes patients with atherosclerosis and patients with CAD than that of healthy controls (Deng, 2016; Saadeldin et al., 2018). It has been reported that overexpression of PGC1α in the skeletal muscle leads to increased secretion of irisin (Bostrom et al., 2012), and reduces the atherosclerotic plaque area by 40% (Shimba et al., 2019). Further, overexpression of PGC1α in the skeletal muscle decreases the vascular cell adhesion moleculer-1 (VCAM-1) and monocyte chemoattractant protein-1 (MCP-1) mRNA and protein expression of aorta in apolipoprotein E-knockout (ApoE-KO) mice (Shimba et al., 2019). Moreover, the treatment of the human umbilical vein endothelial cells (HUVEC) with PGC1a dependent myokines, including irisin and β-aminoisobutyric acid, inhibits the expression of VCAM-1 gene and protein induced by tumor necrosis factor a (TNF-a; Shimba et al., 2019), indicating that irisin is involved in the protective effect of atherosclerosis.

Furthermore, data from animal studies have shown that irisin has a direct therapeutic effect on atherosclerosis (Zhang et al., 2016b; Li et al., 2019). For instance, irisin can ameliorate hyperlipidemia (Li et al., 2019) and promote the proliferation of endothelial cells (ECs) in atherosclerotic mice (Zhang et al., 2016b). In diabetic mice, irisin treatment can significantly ameliorate the endothelial dysfunction and reduce endothelial apoptosis and atherosclerotic plaque area (Lu et al., 2015a). In addition, irisin treatment (0.5 μg/g body weight/day) inhibits the formation of carotid neointima, alleviates aortic inflammation and apoptosis and significantly reduces atherosclerosis in ApoE-deficient mice fed with high cholesterol diet (Zhang et al., 2016a). In the cell model of atherosclerosis, the application of irisin can improve the survival of ECs, promote their migration and tube forming capacity and inhibit apoptosis, proinflammatory cytokine secretion, and reactive oxygen species (ROS) production *via* pAkt/mTOR/nuclear factor E2-related factor-2 (Nrf2) pathway (Zhang et al., 2019).

#### Myocardial Infarction

Myocardial infarction (MI) is characterized by sudden ischemia, resulting in insufficient oxygen supply and damage or death of cardiomyocytes (Lu et al., 2015b). More than half of the deaths caused by cardiovascular diseases are caused by MI (Pollard, 2000). A large number of studies have demonstrated that exercise has a potential role in reducing the occurrence of MI, and in promoting rehabilitation after MI, which has been a common method to improve cardiovascular health. According to the reports, serum irisin concentrations of MI rats increased after 8 weeks of swimming (Bashar et al., 2018). Further, serum irisin concentrations are positive correlated with QRS duration, total antioxidant status, whereas negative correlated with ST elevation, creatine phosphokinase, collagen deposition and caspase-3 expression (Bashar et al., 2018). This indicates that rising serum irisin concentrations *via* regular exercise or taking irisin as a supplement should improve recovery following MI. Indeed, it has been reported that irisin treatment for 2 weeks significantly alleviates cardiac dysfunction and ventricular dilation and reduces infarct size as well as fibrosis induced by MI (Liao et al., 2019). Additionally, *vitro* experiments revealed that the molecular mechanism of the effect mentioned above was related to the angiogenesis *via* activating of extracellular signal-related kinase (ERK) signaling pathway in ECs (Liao et al., 2019).

Ischemia and reperfusion (I/R) and hypoxia-reoxygenation (HR) are commonly used models of MI *in vivo* and *in vitro*, respectively. According to the reports, irisin treatment (100 mg/kg) significantly improves ventricular function recovery and reduces the myocardial infarct size after I/R (Wang et al., 2017). Furthermore, HR experiments of H9C2 cardiomyoblasts further reveal that irisin inhibits the opening of mitochondrial permeability transition pore (mPTP), attenuates mitochondrial swelling, and protects mitochondrial functions, which has become an approach of myocardial protection after MI (Wang et al., 2017). It was reported that HR-induced augmented apoptotic ratio of cardiomyocytes under high glucose stress, whereas irisin treatment increased the activity of AMPK pathway, thereby reducing cellular redox stress, maintaining mitochondria potential, and ultimately protecting cardiomyocytes from HR damage (Fan et al., 2020). Moreover, irisin application at a concentration of 50 nmol/L increased metabolism and differentiation of H9C2 cardiomyocytes by activating expression of exercise related genes, including myocardin, follistatin, and nuclear respiratory factor-1 (Xie et al., 2015). In addition, insufficient autophagy flow is an important pathological factor leading to cardiac remodeling and heart failure. It has been illustrated that overexpression of irisin improves mitophagy, autophagy flow and protects cardiomyocytes from myocardial hypertrophy (Li et al., 2018). Recently, both *in vivo* and *in vitro* studies have revealed that optic atrophy 1 (Opa1) expression is downregulated in infarcted heart, whereas irisin treatment upregulated the expression of Opa1 and protects cardiomyocytes from further damage following MI (Xin and Lu, 2020).

#### Hypertension

It is well-known that hypertension is a severe public health challenge. Data have shown that the prevalence of hypertension ranged from 13 to 41% among 182 countries in the world, accounting for approximately 45% of the global cardiovascular morbidity and mortality (Zeng et al., 2020). Exercise is one of the major non-pharmacological treatments for hypertension, and is broadly recommended by European and American hypertension guidelines (Dimeo et al., 2012). It has been demonstrated that 8 weeks of moderate aerobic exercise training (60–70% VO_2_max), 45 min per time and 3 days per week, reduces the carotid-femoral pulse wave velocity (cfPWV) and arterial stiffness in obese rats (Inoue et al., 2019). Further cellular experiments have revealed that the improvement of vascular function produced by exercise is caused by the activation of AMPK-Akt-endothelial nitric oxide synthase (eNOS) signaling pathway *via* irisin (Inoue et al., 2019). Furthermore, irisin application by intravenous injection (0.1, 1, and 10 μg/kg) decreased the blood pressure of spontaneously hypertensive rats (SHRs) in a concentration-dependent manner but failed in normal Wistar-Kyoto rats (Fu et al., 2016). Further, it has been demonstrated that irisin itself has no direct vasodilation effect on the mesenteric artery of SHRs pretreated with phenylephrine, but it increases the phosphorylation of eNOS and the production of NO in ECs by activating AMPK, thus enhancing the relaxation of mesenteric artery in SHRs induced by acetylcholine (Fu et al., 2016). According to another report, irisin stimulates the Ca^2+^ influx through the transient receptor potential vanilloid subtype 4 (TRPV4) channel, the most important Ca^2+^ permeable cation channels in vascular ECs. As a result, Ca^2+^ influx induces endothelium-dependent vasodilation of rat mesenteric artery (Ye et al., 2018). In addition, recent studies have shown that the antihypertensive effect of exercise may be related to the regulation of central nervous system. It has been found that irisin application can activate Nrf2 in the paraventricular nucleus (PVN) of SHRs, thereby reducing oxidative stress, restoring imbalance of neurotransmitters, and ultimately decreasing blood pressure (Huo et al., 2020). Nevertheless, when Nrf2 is knockdown, the protective effects of irisin on hypertension are abolished (Huo et al., 2020).

Vascular endothelial cell homeostasis is essential for maintaining normal blood pressure. A large number of studies have shown that irisin plays a key role in maintaining endothelial cell homeostasis. Endothelial progenitor cells (EPCs) are precursor cells of the vascular endothelial cells (Diaz Del Moral et al., 2020) and play an important role in maintaining endothelial cell homeostasis and repairing vascular injury. According to reports, intraperitoneal injection of irisin in diabetic mice induced an increase in the number of circulating EPCs (Zhu et al., 2016). Further, the co-culture of irisin promoted the proliferation and the migration of EPCs from the bone narrow of diabetic mice (Zhu et al., 2016). The subsequent experiments revealed that the homeostasis of ECs produced by irisin at least partly *via* activating PI3K/Akt pathway and following increased eNOS expression and phosphorylation (Zhu et al., 2016). Moreover, irisin treatment has been found to promote angiogenesis *via* increasing migration and tube formation, and attenuate chemically-induced vessel angiogenic impairment in zebrafish (Wu et al., 2015). The experiments *in vitro* further confirmed that irisin-induced endothelial homeostasis by activating the ERK signaling pathway (Wu et al., 2015). Additionally, other studies have shown that the activation of the ERK signaling pathway in HUVEC inhibits the expression of Bax and Caspase, thereby promoting cell proliferation and reducing cell apoptosis that was induced by high glucose (Song et al., 2014).

#### Vascular Inflammation

It has been illustrated that physical exercise not only increases the release of irisin, but also inhibits the secretion of pro-inflammatory cytokines and alleviates the inflammatory response of diseases (Mazur-Bialy, 2017). In Chinese obese children, irisin concentrations are significantly lower than that of normal children, and negatively correlated with inflammatory markers of endothelial activation, including high-sensitivity C-reactive protein (hs-CRP), intercellular cell adhesion molecule-1 (ICAM-1) and E-selectin with irisin levels (Yin et al., 2020). This suggests that lower irisin concentrations may indicate an early state of vascular inflammation in obese children. Additionally, after family-based lifestyle intervention, which mainly including aerobic exercise of 5–7 times per week for at least 30 min and dietary recommendations, irisin concentrations of the obese children were significantly increased and following with improvement of cardiovascular and inflammatory parameters (Yin et al., 2020). Furthermore, lower serum irisin concentrations are also found in children with metabolic syndrome, and the irisin concentrations are negatively correlated with biomarkers of endothelial inflammation and dysfunction, such as ICAM-2 and vascular cell adhesion molecule-1 (VCAM-1; Huerta-Delgado et al., 2020). However, other research have found that there is no correlation between serum irisin concentrations and hs-CRP levels in T2D patients (Elizondo-Montemayor et al., 2019). More than that, there is a positive correlation between serum irisin concentrations and hs-CRP in patients with severe inflammation (Buscemi et al., 2020). Given divergences of the correlation between irisin and inflammatory cytokines, some scholars have hypothesized that there is an irisin “pro-inflammatory/anti-inflammatory” axis in the body (Elizondo-Montemayor et al., 2019), which may be indirectly confirmed by other studies. According to the reports of Mazur-Bialy et al. (2017), irisin treatment induced a decrease in the expression of inflammatory cytokines, such as tumor necrosis factor-a (TNF-a), IL-1β, and MCP-1, in murine macrophages. On the other hand, inflammatory factors such as TNF-a and IL-1β can in turn inhibit the expression of FNDC5 in murine myotubes, thereby reducing the secretion of irisin (Matsuo et al., 2015). Therefore, we speculate that when mild inflammation occurs, the secretion of irisin will increase to resist inflammation, thereby inhibiting the level of inflammatory cytokines, which in turn induces a weakened suppression of FNDC5 expression, and further increases the secretion of irisin, resulting in circulating irisin concentration increasing. For example, the circulating irisin concentrations are higher in some obese people who are considered to have low-grade chronic inflammation (Crujeiras et al., 2014; Pardo et al., 2014), and are positively correlated with BMI or body fat percentage (Perakakis et al., 2017; Abulmeaty et al., 2020). Alternatively, when inflammation gets worse, inflammatory cytokines will directly inhibit the expression of FNDC5, resulting in the decrease of circulating irisin concentration, such as in some other obese, diabetic, or severely inflammatory patients (Shoukry et al., 2016; Elizondo-Montemayor et al., 2019; Yin et al., 2020).

As described above, it has been confirmed that irisin is involved in the regulation of inflammation. Since the molecular mechanism has not been fully elucidated, many studies have been conducted to reveal the anti-inflammatory mechanism of irisin. It has been found that high-fat diet (HFD) can induce over-accumulation of perivascular adipose tissue (PVAT), and severely damage the vascular endothelial function. While, irisin treatment significantly alleviated vascular endothelial injury, promoted the production of NO in PVAT, and inhibited the expression of TNF-a (Hou et al., 2017). Furthermore, irisin treatment significantly ameliorated atherosclerosis in ApoE-deficient mice fed on high cholesterol diet and reduced inflammation of the aortic tissue (Zhang et al., 2016b). Moreover, irisin has been found to reverse oxidative stress and inflammation induced by advanced glycation end products (AGEs) in a *vitro* experiment (Deng et al., 2018). Meanwhile, other studies have found that the anti-inflammatory effect of irisin is related to the activation of AMPK-Phosphatidylinositol-4,5-bisphosphate 3-kinase (PI3K)-Akt-eNOS pathway (Lu et al., 2015a), or the inhibition of the ROS/p38 MAPK/NF-κB pathway in HUVECs (Zhang et al., 2016a). Additionally, it was demonstrated that FNDC5, the irisin precursor, induced the decrease of M1 polarization in macrophages and thus suppressed the pro-inflammatory cytokine expression (Xiong et al., 2018). Furthermore, irisin was also reported to convert adipose tissue macrophage polarization from M1 pro-inflammatory phenotype to M2 anti-inflammatory phenotype (Dong et al., 2016). Irisin has also been found to reduce the overproduction of reactive oxygen species (ROS) in a concentration-dependent manner, thereby regulating the activity and phagocytosis of macrophages, and exerting potential anti-inflammatory properties (Mazur-Bialy et al., 2017).

**Figure 2:**
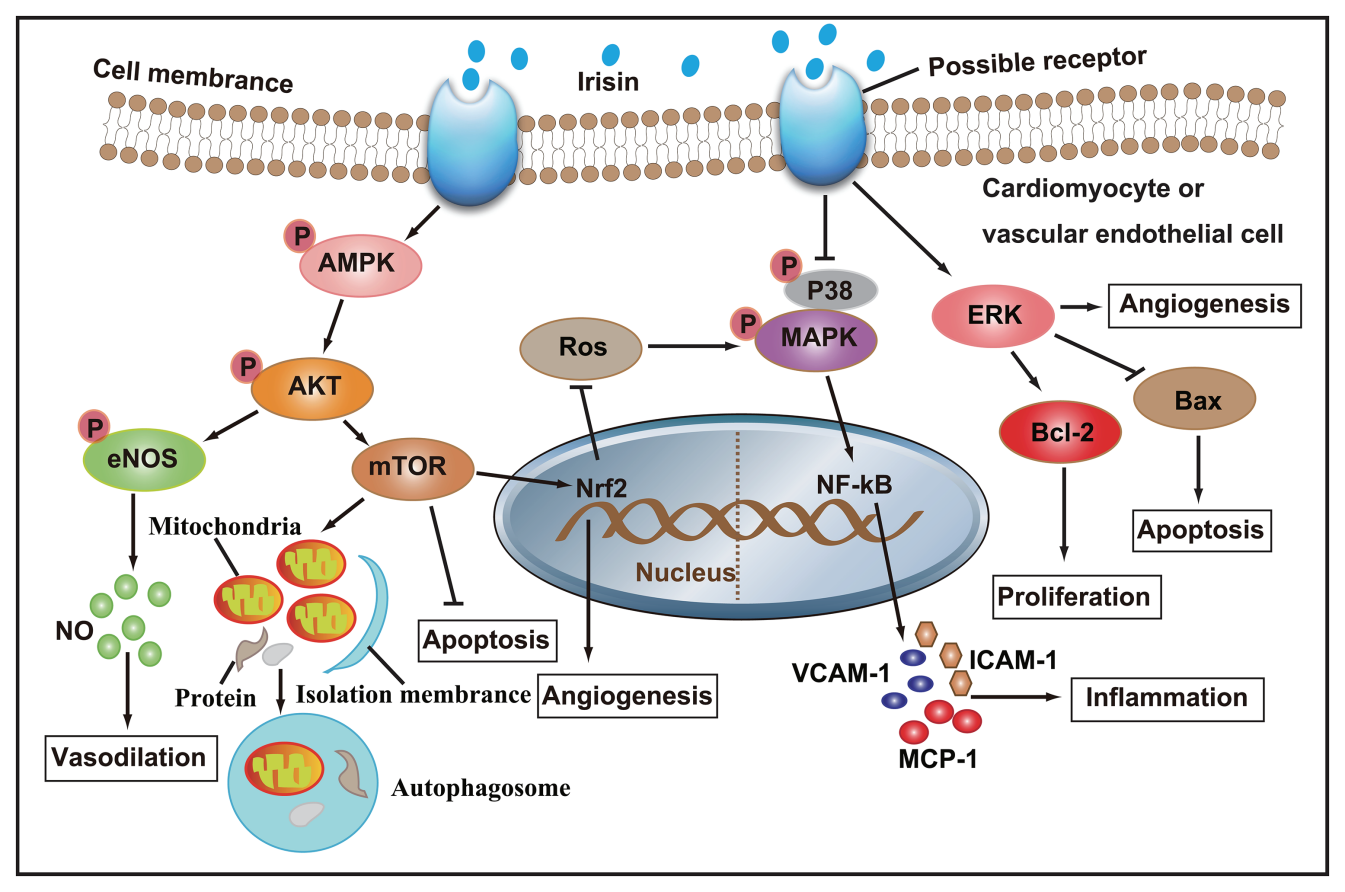
After irisin binds to membrane receptors, AMPK phosphorylation increase, and its downstream Akt/eNOS and Akt/mTOR signaling cascades are activated, thereby promoting NO secretion, autophagy, and angiogenesis, and inhibiting apoptosis and ROS production. On the other hand, irisin can also inhibit the secretion of pro-inflammatory factors by inhibiting p38 MAPK signaling pathway, and promote angiogenesis and cell proliferation, and inhibit apoptosis by activating ERK signaling pathway.

Taken together, studies both *in vivo* and *in vitro* have confirmed that irisin participates in the regulation of cardiovascular health. A series of molecular mechanisms, such as NO production, autophagy, angiogenesis, and inflammation in cardiomyocytes or vascular ECs, have been involved in the regulation of irisin in cardiovascular diseases (Figure 2). Based on the theoretical basis of irisin, formulating scientific exercise prescriptions is of great significance to the prevention of cardiovascular disease. Clinically, the injection of irisin in the treatment of cardiovascular disease will have a good prospect.

## Conclusion and Prospects

**Figure 3:**
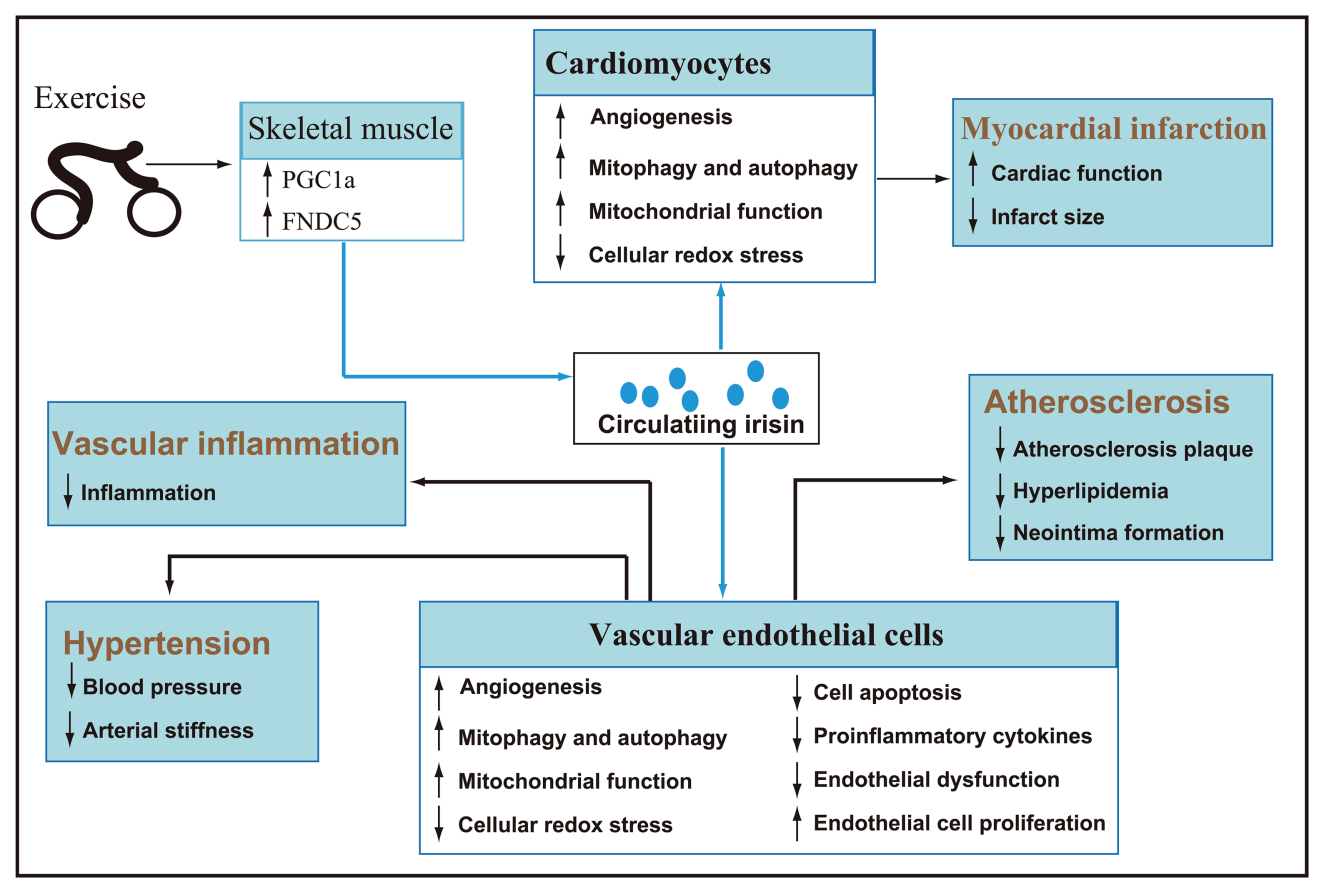
After secreted by skeletal muscle during exercise, irisin reaches cardiomyocytes and vascular endothelial cells *via* blood circulation (blue line). Irisin can promote angiogenesis, autophagy, and endothelial cell proliferation, improve mitochondrial function, inhibit cellular redox stress, apoptosis, inflammation, and endothelial dysfunction, and then play a key role in improving hypertension, myocardial infarction, atherosclerosis, and vascular inflammation.

As a hormone mainly secreted by skeletal muscle, irisin is regulated by exercise. Acute exercise can increase the concentration of circulating irisin, and chronic exercise can improve irisin metabolic dynamic and selectively increase the circulating irisin concentration of subjects. Given the significant role in the browning of white fat and energy metabolism, the research of irisin mainly focuses on metabolic diseases. Recently, an emerging number of animal and clinical trials have confirmed that irisin is involved in the regulation of cardiovascular health and has potential therapeutic effects (Figure 3). However, there are inconsistencies in the research and literature reports, and the detailed mechanism of irisin in promoting cardiovascular health under pathological conditions has not been fully elucidated. Therefore, a lot of work needs to be done in the future to solve the problems.

First, as described previously, exercise intensity is an important factor affecting the metabolic dynamics and concentrations of circulating irisin. We could not help wondering that what is the intensity threshold? Are there different intensity thresholds for different exercise types, for example, aerobic exercise and resistance exercise? Will chronic exercise improve irisin sensitivity the same way as improve insulin sensitivity, so that the baseline irisin concentrations of people who exercise regularly will not increase? Answering the above questions will help us to formulate scientific exercise prescriptions for rehabilitation treatment and cardiovascular diseases.

Second, it is urgent to develop more accurate clinical testing methods. It has been reported that the difference between the test results of the two most commonly used ELISA kits is about 10 times (Huh et al., 2015), and the available commercial polyclonal antibodies produce multiple immune bands due to poor specificity, whereas these immune bands cannot be irisin (Albrecht et al., 2015, 2020). Reliable assays and precise design will help us to realize the practice of irisin’s clinical diagnosis of cardiovascular health in the future.

Third, it has been confirmed that αVβ5 integrin is the irisin receptor of osteocytes, adipocytes and enterocytes (Kim et al., 2019; Bi et al., 2020). Given the effective regulation of irisin on the functions of cardiomyocytes and vascular ECs, whether αVβ5 integrin is also the irisin receptor of these cells is the direction of future research. The further identification of irisin receptors in cardiomyocytes and vascular ECs will open up new approaches for the treatment of cardiovascular diseases.

## Author Contributions

CM and HD prepared the first draft and wrote the final version of the manuscript. YD and HL were involved in literature search. XX and YY critically revised the manuscript and gave constructive opinions on the article. All authors contributed to the article and approved the submitted version.

### Conflict of Interest

The authors declare that the research was conducted in the absence of any commercial or financial relationships that could be construed as a potential conflict of interest.

## References

[ref1] Abulmeaty M. M. A. Almajwal A. M. Alam I. Razak S. ElSadek M. F. Aljuraiban G. S.. (2020). Relationship of vitamin D-deficient diet and irisin, and their impact on energy homeostasis in rats. Front. Physiol. 11:25. 10.3389/fphys.2020.00025, PMID: 32082189 PMC7005576

[ref2] Albrecht E. Norheim F. Thiede B. Holen T. Ohashi T. Schering L.. (2015). Irisin - a myth rather than an exercise-inducible myokine. Sci. Rep. 5:8889. 10.1038/srep08889, PMID: 25749243 PMC4352853

[ref3] Albrecht E. Schering L. Buck F. Vlach K. Schober H. C. Drevon C. A.. (2020). Irisin: still chasing shadows. Mol. Metab. 34, 124–135. 10.1016/j.molmet.2020.01.016, PMID: 32180552 PMC7033458

[ref4] Angosta A. D. Reyes A. T. Cross C. Pollom T. Sood K. (2020). Cardiovascular disease knowledge, risk factors, and resilience among US veterans with and without post-traumatic stress disorder. J. Am. Assoc. Nurse Pract. 10.1097/JXX.0000000000000507 [Epub ahead of print] 32976251

[ref5] Aydin S. Aydin S. Kobat M. A. Kalayci M. Eren M. N. Yilmaz M.. (2014). Decreased saliva/serum irisin concentrations in the acute myocardial infarction promising for being a new candidate biomarker for diagnosis of this pathology. Peptides 56, 141–145. 10.1016/j.peptides.2014.04.002, PMID: 24747283

[ref6] Aydin S. Aydin S. Kuloglu T. Yilmaz M. Kalayci M. Sahin I.. (2013). Alterations of irisin concentrations in saliva and serum of obese and normal-weight subjects, before and after 45 min of a Turkish bath or running. Peptides 50, 13–18. 10.1016/j.peptides.2013.09.011, PMID: 24096106

[ref7] Bashar S. M. Samir El-Sherbeiny S. M. Boraie M. Z. (2018). Correlation between the blood level of irisin and the severity of acute myocardial infarction in exercise-trained rats. J. Basic Clin. Physiol. Pharmacol. 30, 59–71. 10.1515/jbcpp-2018-0090, PMID: 30265651

[ref8] Bell M. A. Levine C. B. Downey R. L. Griffitts C. Mann S. Frye C. W.. (2016). Influence of endurance and sprinting exercise on plasma adiponectin, leptin and irisin concentrations in racing Greyhounds and sled dogs. Aust. Vet. J. 94, 154–159. 10.1111/avj.12436, PMID: 27113986

[ref9] Benjamin E. J. Blaha M. J. Chiuve S. E. Cushman M. Das S. R. Deo R.. (2017). Heart disease and stroke statistics-2017 update: a report from the American Heart Association. Circulation 135, e146–e603. 10.1161/CIR.0000000000000485, PMID: 28122885 PMC5408160

[ref10] Bi J. Zhang J. Ren Y. Du Z. Li T. Wang T.. (2020). Irisin reverses intestinal epithelial barrier dysfunction during intestinal injury via binding to the integrin alphaVbeta5 receptor. J. Cell. Mol. Med. 24, 996–1009. 10.1111/jcmm.14811, PMID: 31701659 PMC6933384

[ref11] Bostrom P. Wu J. Jedrychowski M. P. Korde A. Ye L. Lo J. C.. (2012). A PGC1-alpha-dependent myokine that drives brown-fat-like development of white fat and thermogenesis. Nature 481, 463–468. 10.1038/nature10777, PMID: 22237023 PMC3522098

[ref12] Buscemi S. Corleo D. Vasto S. Buscemi C. Barile A. M. Rosafio G., et al. (2020). Serum irisin concentrations in severely inflamed patients. Horm. Metab. Res. 52, 246–250. 10.1055/a-1111-9249, PMID: 32079027

[ref13] Cooper C. Moon H. Y., and van Praag H. (2018). On the run for hippocampal plasticity. Cold Spring Harb. Perspect. Med. 8:a029736. 10.1101/cshperspect.a029736, PMID: 28495803 PMC5880155

[ref14] Crujeiras A. B. Zulet M. A. Lopez-Legarrea P. de la Iglesia R. Pardo M. Carreira M. C.. (2014). Association between circulating irisin levels and the promotion of insulin resistance during the weight maintenance period after a dietary weight-lowering program in obese patients. Metabolism 63, 520–531. 10.1016/j.metabol.2013.12.007, PMID: 24439241

[ref15] Deng W. (2016). Association of serum irisin concentrations with presence and severity of coronary artery disease. Med. Sci. Monit. 22, 4193–4197. 10.12659/MSM.897376, PMID: 27815563 PMC5100837

[ref16] Deng X. Huang W. Peng J. Zhu T. T. Sun X. L. Zhou X. Y.. (2018). Irisin alleviates advanced glycation end products-induced inflammation and endothelial dysfunction via inhibiting ROS-NLRP3 inflammasome signaling. Inflammation 41, 260–275. 10.1007/s10753-017-0685-3, PMID: 29098483

[ref17] Diabetes A. A. O. (2012). Diabetes and physical activity. Diabetes Educ. 38, 129–132. 10.1177/0145721711426094 22328039

[ref18] Diaz Del Moral S. Barrena S. Munoz-Chapuli R. Carmona R. (2020). Embryonic circulating endothelial progenitor cells. Angiogenesis 23, 531–541. 10.1007/s10456-020-09732-y, PMID: 32613361

[ref19] Dimeo F. Pagonas N. Seibert F. Arndt R. Zidek W. Westhoff T. H. (2012). Aerobic exercise reduces blood pressure in resistant hypertension. Hypertension 60, 653–658. 10.1161/HYPERTENSIONAHA.112.197780, PMID: 22802220

[ref20] Dong J. Dong Y. Dong Y. Chen F. Mitch W. E. Zhang L. (2016). Inhibition of myostatin in mice improves insulin sensitivity via irisin-mediated cross talk between muscle and adipose tissues. Int. J. Obes. 40, 434–442. 10.1038/ijo.2015.200, PMID: 26435323 PMC4783239

[ref21] Efe T. H. Acar B. Ertem A. G. Yayla K. G. Algul E. Yayla C.. (2017). Serum irisin level can predict the severity of coronary artery disease in patients with stable angina. Korean Circ. J. 47, 44–49. 10.4070/kcj.2016.0079, PMID: 28154590 PMC5287186

[ref22] Elizondo-Montemayor L. Gonzalez-Gil A. M. Tamez-Rivera O. Toledo-Salinas C. Peschard-Franco M. Rodríguez-Gutiérrez N. A.. (2019). Association between irisin, hs-CRP, and metabolic status in children and adolescents with type 2 diabetes mellitus. Mediat. Inflamm. 2019, 1–13. 10.1155/2019/6737318, PMID: 31015797 PMC6446111

[ref23] Elsen M. Raschke S. Eckel J. (2014). Browning of white fat: does irisin play a role in humans? J. Endocrinol. 222, R25–R38. 10.1530/JOE-14-0189, PMID: 24781257

[ref24] Erickson H. P. (2013). Irisin and FNDC5 in retrospect: an exercise hormone or a transmembrane receptor? Adipocytes 2, 289–293. 10.4161/adip.26082, PMID: 24052909 PMC3774709

[ref25] Fain J. N. Company J. M. Booth F. W. Laughlin M. H. Padilla J. Jenkins N. T.. (2013). Exercise training does not increase muscle FNDC5 protein or mRNA expression in pigs. Metabolism 62, 1503–1511. 10.1016/j.metabol.2013.05.021, PMID: 23831442 PMC3779497

[ref26] Fan J. Zhu Q. Wu Z. Ding J. Qin S. Liu H.. (2020). Protective effects of irisin on hypoxia-reoxygenation injury in hyperglycemia-treated cardiomyocytes: role of AMPK pathway and mitochondrial protection. J. Cell. Physiol. 235, 1165–1174. 10.1002/jcp.29030, PMID: 31268170

[ref27] Fatouros I. G. (2018). Is irisin the new player in exercise-induced adaptations or not? A 2017 update. Clin. Chem. Lab. Med. 56, 525–548. 10.1515/cclm-2017-0674, PMID: 29127759

[ref28] Fiuza-Luces C. Santos-Lozano A. Joyner M. Carrera-Bastos P. Picazo O. Zugaza J. L.. (2018). Exercise benefits in cardiovascular disease: beyond attenuation of traditional risk factors. Nat. Rev. Cardiol. 15, 731–743. 10.1038/s41569-018-0065-1, PMID: 30115967

[ref29] Fox J. Rioux B. V. Goulet E. D. B. Johanssen N. M. Swift D. L. Bouchard D. R.. (2018). Effect of an acute exercise bout on immediate post-exercise irisin concentration in adults: a meta-analysis. Scand. J. Med. Sci. Sports 28, 16–28. 10.1111/sms.12904, PMID: 28453881

[ref30] Fu J. Han Y. Wang J. Liu Y. Zheng S. Zhou L.. (2016). Irisin lowers blood pressure by improvement of endothelial dysfunction via AMPK-Akt-eNOS-NO pathway in the spontaneously hypertensive rat. J. Am. Heart Assoc. 5:e003433. 10.1161/JAHA.116.003433, PMID: 27912206 PMC5210324

[ref31] Gmiat A. Mieszkowski J. Prusik K. Prusik K. Kortas J. Kochanowicz A.. (2017). Changes in pro-inflammatory markers and leucine concentrations in response to Nordic Walking training combined with vitamin D supplementation in elderly women. Biogerontology 18, 535–548. 10.1007/s10522-017-9694-8, PMID: 28316011 PMC5514208

[ref32] Greenhill C. (2019). Irisin receptor in osteocytes identified. Nat. Rev. Endocrinol. 15:63. 10.1038/s41574-018-0151-9, PMID: 30602738

[ref33] Guo W. Zhang B. Wang X. (2020). Lower irisin levels in coronary artery disease: a meta-analysis. Minerva Endocrinol. 45, 61–69. 10.23736/S0391-1977.17.02663-3, PMID: 29160049

[ref34] Hecksteden A. Wegmann M. Steffen A. Kraushaar J. Morsch A. Ruppenthal S.. (2013). Irisin and exercise training in humans-results from a randomized controlled training trial. BMC Med. 11:235. 10.1186/1741-7015-11-235, PMID: 24191966 PMC4228275

[ref35] Hisamatsu T. Miura K. Arima H. Fujiyoshi A. Kadota A. Kadowaki S.. (2018). Relationship of serum irisin levels to prevalence and progression of coronary artery calcification: a prospective, population-based study. Int. J. Cardiol. 267, 177–182. 10.1016/j.ijcard.2018.05.075, PMID: 29859711

[ref36] Hofmann T. Elbelt U. Stengel A. (2014). Irisin as a muscle-derived hormone stimulating thermogenesis—a critical update. Peptides 54, 89–100. 10.1016/j.peptides.2014.01.016, PMID: 24472856

[ref37] Hou N. Du G. Han F. Zhang J. Jiao X. Sun X. (2017). Irisin regulates heme oxygenase-1/adiponectin axis in perivascular adipose tissue and improves endothelial dysfunction in diet-induced obese mice. Cell. Physiol. Biochem. 42, 603–614. 10.1159/000477864, PMID: 28595178

[ref38] Huerta-Delgado A. S. Roffe-Vazquez D. N. Gonzalez-Gil A. M. Villarreal-Calderon J. R. Tamez-Rivera O. Rodriguez-Gutierrez N. A.. (2020). Serum irisin levels, endothelial dysfunction, and inflammation in pediatric patients with Type 2 diabetes mellitus and metabolic syndrome. J. Diabetes Res. 2020:1949415. 10.1155/2020/1949415, PMID: 32964051 PMC7492943

[ref39] Huh J. Y. Mougios V. Skraparlis A. Kabasakalis A. Mantzoros C. S. (2014). Irisin in response to acute and chronic whole-body vibration exercise in humans. Metabolism 63, 918–921. 10.1016/j.metabol.2014.04.001, PMID: 24814685

[ref40] Huh J. Y. Panagiotou G. Mougios V. Brinkoetter M. Vamvini M. T. Schneider B. E.. (2012). FNDC5 and irisin in humans: I. predictors of circulating concentrations in serum and plasma and II. mRNA expression and circulating concentrations in response to weight loss and exercise. Metabolism 61, 1725–1738. 10.1016/j.metabol.2012.09.002, PMID: 23018146 PMC3614417

[ref41] Huh J. Y. Siopi A. Mougios V. Park K. H. Mantzoros C. S. (2015). Irisin in response to exercise in humans with and without metabolic syndrome. J. Clin. Endocrinol. Metab. 100, E453–E457. 10.1210/jc.2014-2416, PMID: 25514098

[ref42] Huo C. J. Yu X. J. Sun Y. J. Li H. B. Su Q. Bai J.. (2020). Irisin lowers blood pressure by activating the Nrf2 signaling pathway in the hypothalamic paraventricular nucleus of spontaneously hypertensive rats. Toxicol. Appl. Pharmacol. 394:114953. 10.1016/j.taap.2020.114953, PMID: 32165127

[ref43] Icli A. Cure E. Cumhur Cure M. Uslu A. U. Balta S. Arslan S.. (2016). Novel myokine: irisin may be an independent predictor for subclinic atherosclerosis in Behcet’s disease. J. Investig. Med. 64, 875–881. 10.1136/jim-2015-000044, PMID: 26941246 PMC4819671

[ref44] Inoue K. Fujie S. Hasegawa N. Horii N. Uchida M. Iemitsu K.. (2019). Aerobic exercise training-induced irisin secretion is associated with the reduction of arterial stiffness via nitric oxide production in adults with obesity. Appl. Physiol. Nutr. Metab. 45, 715–722. 10.1139/apnm-2019-0602, PMID: 31860334

[ref45] Ivanov I. P. Firth A. E. Michel A. M. Atkins J. F. Baranov P. V. (2011). Identification of evolutionarily conserved non-AUG-initiated N-terminal extensions in human coding sequences. Nucleic Acids Res. 39, 4220–4234. 10.1093/nar/gkr007, PMID: 21266472 PMC3105428

[ref46] Jedrychowski M. P. Wrann C. D. Paulo J. A. Gerber K. K. Szpyt J. Robinson M. M.. (2015). Detection and quantitation of circulating human irisin by tandem mass spectrometry. Cell Metab. 22, 734–740. 10.1016/j.cmet.2015.08.001, PMID: 26278051 PMC4802359

[ref47] Kim H. Wrann C. D. Jedrychowski M. Vidoni S. Kitase Y. Nagano K.. (2019). Irisin mediates effects on bone and fat via αV integrin receptors. Cell 178, 507–508. 10.1016/j.cell.2019.06.028, PMID: 31299203 PMC6707723

[ref48] Kurdiova T. Balaz M. Vician M. Maderova D. Vlcek M. Valkovic L.. (2014). Effects of obesity, diabetes and exercise on Fndc5 gene expression and irisin release in human skeletal muscle and adipose tissue: in vivo and in vitro studies. J. Physiol. 592, 1091–1107. 10.1113/jphysiol.2013.264655, PMID: 24297848 PMC3948565

[ref49] Lee P. Linderman J. D. Smith S. Brychta R. J. Wang J. Idelson C.. (2014). Irisin and FGF21 are cold-induced endocrine activators of brown fat function in humans. Cell Metab. 19, 302–309. 10.1016/j.cmet.2013.12.017, PMID: 24506871 PMC7647184

[ref50] Li H. Shen J. Wu T. Kuang J. Liu Q. Cheng S.. (2019). Irisin is controlled by farnesoid X receptor and regulates cholesterol homeostasis. Front. Pharmacol. 10:548. 10.3389/fphar.2019.01702, PMID: 31191305 PMC6546903

[ref51] Li R. -L. Wu S. -S. Wu Y. Wang X. -X. Chen H. -Y. Xin J. -J.. (2018). Irisin alleviates pressure overload-induced cardiac hypertrophy by inducing protective autophagy via mTOR-independent activation of the AMPK-ULK1 pathway. J. Mol. Cell. Cardiol. 121, 242–255. 10.1016/j.yjmcc.2018.07.250, PMID: 30053525

[ref52] Liao Q. Qu S. Tang L. X. Li L. P. He D. F. Zeng C. Y., et al. (2019). Irisin exerts a therapeutic effect against myocardial infarction via promoting angiogenesis. Acta Pharmacol. Sin. 40, 1314–1321. 10.1038/s41401-019-0230-z, PMID: 31061533 PMC6786355

[ref53] Lourenco M. V. Frozza R. L. de Freitas G. B. Zhang H. Kincheski G. C. Ribeiro F. C.. (2019). Exercise-linked FNDC5/irisin rescues synaptic plasticity and memory defects in Alzheimer’s models. Nat. Med. 25, 165–175. 10.1038/s41591-018-0275-4, PMID: 30617325 PMC6327967

[ref55] Lu L. Liu M. Sun R. Zheng Y. Zhang P. (2015b). Myocardial infarction: symptoms and treatments. Cell Biochem. Biophys. 3, 865–867. 10.1007/s12013-015-0553-4, PMID: 25638347

[ref54] Lu J. Xiang G. Liu M. Mei W. Xiang L. Dong J. (2015a). Irisin protects against endothelial injury and ameliorates atherosclerosis in apolipoprotein E-null diabetic mice. Atherosclerosis 243, 438–448. 10.1016/j.atherosclerosis.2015.10.020, PMID: 26520898

[ref56] Mai S. Grugni G. Mele C. Vietti R. Vigna L. Sartorio A.. (2020). Irisin levels in genetic and essential obesity: clues for a potential dual role. Sci. Rep. 10:1020. 10.1038/s41598-020-57855-5, PMID: 31974460 PMC6978420

[ref57] Matsuo Y. Gleitsmann K. Mangner N. Werner S. Fischer T. Bowen T. S.. (2015). Fibronectin type III domain containing 5 expression in skeletal muscle in chronic heart failure-relevance of inflammatory cytokines. J. Cachexia Sarcopenia Muscle 6, 62–72. 10.1002/jcsm.12006, PMID: 26136413 PMC4435098

[ref58] Mazur-Bialy A. I. (2017). Irisin acts as a regulator of macrophages host defense. Life Sci. 176, 21–25. 10.1016/j.lfs.2017.03.011, PMID: 28315350

[ref59] Mazur-Bialy A. I. Bilski J. Pochec E. Brzozowski T. (2017). New insight into the direct anti-inflammatory activity of a myokine irisin against proinflammatory activation of adipocytes. Implication for exercise in obesity. J. Physiol. Pharmacol. 68, 243–251. PMID: 28614774

[ref60] Miyamoto-Mikami E. Sato K. Kurihara T. Hasegawa N. Fujie S. Fujita S., et al. (2015). Endurance training-induced increase in circulating irisin levels is associated with reduction of abdominal visceral fat in middle-aged and older adults. PLoS One 10:e0120354. 10.1371/journal.pone.0120354, PMID: 25793753 PMC4368602

[ref61] Moore S. C. Patel A. V. Matthews C. E. Berrington de Gonzalez A. Park Y. Katki H. A.. (2012). Leisure time physical activity of moderate to vigorous intensity and mortality: a large pooled cohort analysis. PLoS Med. 9:e1001335. 10.1371/journal.pmed.1001335, PMID: 23139642 PMC3491006

[ref62] Moreno M. Moreno-Navarrete J. M. Serrano M. Ortega F. Delgado E. Sanchez-Ragnarsson C.. (2015). Circulating irisin levels are positively associated with metabolic risk factors in sedentary subjects. PLoS One 10:e0124100. 10.1371/journal.pone.0124100, PMID: 25897751 PMC4405583

[ref63] Moreno-Navarrete J. M. Ortega F. Serrano M. Guerra E. Pardo G. Tinahones F., et al. (2013). Irisin is expressed and produced by human muscle and adipose tissue in association with obesity and insulin resistance. J. Clin. Endocrinol. Metab. 98, E769–E778. 10.1210/jc.2012-2749, PMID: 23436919

[ref64] Nguyen M. T. Fernando S. Schwarz N. Tan J. T. Bursill C. A. Psaltis P. J. (2019). Inflammation as a therapeutic target in atherosclerosis. J. Clin. Med. 8:1109. 10.3390/jcm8081109, PMID: 31357404 PMC6722844

[ref65] Ozbay S. Ulupinar S. Sebin E. Altinkaynak K. (2020). Acute and chronic effects of aerobic exercise on serum irisin, adropin, and cholesterol levels in the winter season: indoor training versus outdoor training. Chin. J. Phys. 63, 21–26. 10.4103/CJP.CJP_84_19, PMID: 32056983

[ref66] Panagiotou G. Mu L. Na B. Mukamal K. J. Mantzoros C. S. (2014). Circulating irisin, omentin-1, and lipoprotein subparticles in adults at higher cardiovascular risk. Metabolism 63, 1265–1271. 10.1016/j.metabol.2014.06.001, PMID: 25060690 PMC4175146

[ref67] Pardo M. Crujeiras A. B. Amil M. Aguera Z. Jimenez-Murcia S. Banos R., et al. (2014). Association of irisin with fat mass, resting energy expenditure, and daily activity in conditions of extreme body mass index. Int. J. Endocrinol. 2014:857270. 10.1155/2014/857270, PMID: 24864142 PMC4016898

[ref68] Perakakis N. Triantafyllou G. A. Fernandez-Real J. M. Huh J. Y. Park K. H. Seufert J.. (2017). Physiology and role of irisin in glucose homeostasis. Nat. Rev. Endocrinol. 13, 324–337. 10.1038/nrendo.2016.221, PMID: 28211512 PMC5878942

[ref69] Pollard T. J. (2000). The acute myocardial infarction. Prim. Care 27, 631–649. 10.1016/s0095-4543(05)70167-6, PMID: 10918673

[ref70] Polyzos S. A. Mathew H. Mantzoros C. S. (2015). Irisin: a true, circulating hormone. Metabolism 64, 1611–1618. 10.1016/j.metabol.2015.09.001, PMID: 26422316

[ref71] Raschke S. Elsen M. Gassenhuber H. Sommerfeld M. Schwahn U. Brockmann B.. (2013). Evidence against a beneficial effect of irisin in humans. PLoS One 8:e73680. 10.1371/journal.pone.0073680, PMID: 24040023 PMC3770677

[ref72] Reycraft J. T. Islam H. Townsend L. K. Hayward G. C. Hazell T. J. Macpherson R. E. K. (2020). Exercise intensity and recovery on circulating brain-derived neurotrophic factor. Med. Sci. Sports Exerc. 52, 1210–1217. 10.1249/MSS.0000000000002242, PMID: 31815833

[ref73] Ruan Q. Zhang L. Ruan J. Zhang X. Chen J. Ma C.. (2018). Detection and quantitation of irisin in human cerebrospinal fluid by tandem mass spectrometry. Peptides 103, 60–64. 10.1016/j.peptides.2018.03.013, PMID: 29574076

[ref74] Saadeldin M. K. Elshaer S. S. Emara I. A. Maged M. Abdel-Aziz A. K. (2018). Serum sclerostin and irisin as predictive markers for atherosclerosis in Egyptian type II diabetic female patients: a case control study. PLoS One 13:e0206761. 10.1371/journal.pone.0206761, PMID: 30403705 PMC6221312

[ref75] Samy D. M. Ismail C. A. Nassra R. A. (2015). Circulating irisin concentrations in rat models of thyroid dysfunction—effect of exercise. Metabolism 64, 804–813. 10.1016/j.metabol.2015.01.001, PMID: 25720940

[ref76] Sanchis-Gomar F. Alis R. Pareja-Galeano H. Romagnoli M. Perez-Quilis C. (2014). Inconsistency in circulating irisin levels: what is really happening? Horm. Metab. Res. 46, 591–596. 10.1055/s-0033-1363283, PMID: 24459033

[ref77] Seo D. Y. Bae J. H. Kim T. N. Kwak H. B. Kha P. T. Han J. (2020). Exercise-induced circulating irisin level is correlated with improved cardiac function in rats. Int. J. Environ. Res. Public Health 17:3863. 10.3390/ijerph17113863, PMID: 32485990 PMC7313080

[ref78] Sesti G. Andreozzi F. Fiorentino T. V. Mannino G. C. Sciacqua A. Marini M. A.. (2014). High circulating irisin levels are associated with insulin resistance and vascular atherosclerosis in a cohort of nondiabetic adult subjects. Acta Diabetol. 51, 705–713. 10.1007/s00592-014-0576-0, PMID: 24619655

[ref79] Shen S. Gao R. Bei Y. Li J. Zhang H. Zhou Y.. (2017). Serum irisin predicts mortality risk in acute heart failure patients. Cell. Physiol. Biochem. 42, 615–622. 10.1159/000477867, PMID: 28595171

[ref80] Shimba Y. Togawa H. Senoo N. Ikeda M. Miyoshi N. Morita A.. (2019). Skeletal muscle-specific PGC-1α overexpression suppresses atherosclerosis in apolipoprotein E-knockout mice. Sci. Rep. 9:4077. 10.1038/s41598-019-40643-1, PMID: 30858489 PMC6411944

[ref81] Shoukry A. Shalaby S. M. El-Arabi Bdeer S. Mahmoud A. A. Mousa M. M. Khalifa A. (2016). Circulating serum irisin levels in obesity and type 2 diabetes mellitus. IUBMB Life 68, 544–556. 10.1002/iub.1511, PMID: 27220658

[ref82] Song H. Wu F. Zhang Y. Zhang Y. Wang F. Jiang M.. (2014). Irisin promotes human umbilical vein endothelial cell proliferation through the ERK signaling pathway and partly suppresses high glucose-induced apoptosis. PLoS One 9:e110273. 10.1371/journal.pone.0110273, PMID: 25338001 PMC4206299

[ref83] Spiegelman B. M. Wrann C. (2014). Response to Comment on Wu and Spiegelman. Irisin ERKs the fat. Diabetes 2014;63:381-383. Diabetes 63:e17. 10.2337/db14-0898, PMID: 25146478

[ref84] Timmons J. A. Baar K. Davidsen P. K. Atherton P. J. (2012). Is irisin a human exercise gene? Nature 488, E9–E10. 10.1038/nature11364, PMID: 22932392

[ref85] Tsuchiya Y. Ando D. Goto K. Kiuchi M. Yamakita M. Koyama K. (2014). High-intensity exercise causes greater irisin response compared with low-intensity exercise under similar energy consumption. Tohoku J. Exp. Med. 233, 135–140. 10.1620/tjem.233.135, PMID: 24910199

[ref86] Tsuchiya Y. Ando D. Takamatsu K. Goto K. (2015). Resistance exercise induces a greater irisin response than endurance exercise. Metabolism 64, 1042–1050. 10.1016/j.metabol.2015.05.010, PMID: 26081427

[ref87] Tu W. J. Qiu H. C. Cao J. L. Liu Q. Zeng X. W. Zhao J. Z. (2018). Decreased concentration of irisin is associated with poor functional outcome in ischemic stroke. Neurotherapeutics 15, 1158–1167. 10.1007/s13311-018-0651-2, PMID: 30030698 PMC6277286

[ref88] Wang H. Zhao Y. T. Zhang S. Dubielecka P. M. Du J. Yano N., et al. (2017). Irisin plays a pivotal role to protect the heart against ischemia and reperfusion injury. J. Cell. Physiol. 232, 3775–3785. 10.1002/jcp.25857, PMID: 28181692 PMC5550372

[ref89] Wu F. Song H. Zhang Y. Zhang Y. Mu Q. Jiang M.. (2015). Irisin induces angiogenesis in human umbilical vein endothelial cells in vitro and in zebrafish embryos in vivo via activation of the ERK signaling pathway. PLoS One 10:e0134662. 10.1371/journal.pone.0134662, PMID: 26241478 PMC4524626

[ref90] Wu J. Spiegelman B. M. (2014). Irisin ERKs the fat. Diabetes 63, 381–383. 10.2337/db13-1586, PMID: 24464712 PMC4876758

[ref91] Xie C. Zhang Y. Tran T. D. Wang H. Li S. George E. V.. (2015). Irisin controls growth, intracellular Ca^2+^ signals, and mitochondrial thermogenesis in cardiomyoblasts. PLoS One 10:e0136816. 10.1371/journal.pone.0136816, PMID: 26305684 PMC4549318

[ref92] Xin T. Lu C. (2020). Irisin activates Opa1-induced mitophagy to protect cardiomyocytes against apoptosis following myocardial infarction. Aging 12, 4474–4488. 10.18632/aging.102899, PMID: 32155590 PMC7093202

[ref93] Xiong X. Q. Geng Z. Zhou B. Zhang F. Han Y. Zhou Y. B.. (2018). FNDC5 attenuates adipose tissue inflammation and insulin resistance via AMPK-mediated macrophage polarization in obesity. Metabolism 83, 31–41. 10.1016/j.metabol.2018.01.013, PMID: 29374559

[ref94] Ye L. Xu M. Hu M. Zhang H. Tan X. Li Q.. (2018). TRPV4 is involved in irisin-induced endothelium-dependent vasodilation. Biochem. Biophys. Res. Commun. 495, 41–45. 10.1016/j.bbrc.2017.10.160, PMID: 29097199

[ref95] Yin C. Hu W. Wang M. Lv W. Jia T. Xiao Y. (2020). Irisin as a mediator between obesity and vascular inflammation in Chinese children and adolescents. Nutr. Metab. Cardiovasc. Dis. 30, 320–329. 10.1016/j.numecd.2019.09.025, PMID: 31740239

[ref96] Yuksel Ozgor B. Demiral I. Zeybek U. Celik F. Buyru F. Yeh J.. (2020). Effects of irisin compared with exercise on specific metabolic and obesity parameters in female mice with obesity. Metab. Syndr. Relat. Disord. 18, 141–145. 10.1089/met.2019.0083, PMID: 32250208

[ref97] Zeng Z. Chen J. Xiao C. Chen W. (2020). A global view on prevalence of hypertension and human develop index. Ann. Glob. Health 86:67. 10.5334/aogh.2591, PMID: 32676296 PMC7333558

[ref100] Zhang Y. Li R. Meng Y. Li S. Donelan W. Zhao Y.. (2014). Irisin stimulates browning of white adipocytes through mitogen-activated protein kinase p38 MAP kinase and ERK MAP kinase signaling. Diabetes 63, 514–525. 10.2337/db13-1106, PMID: 24150604 PMC13117908

[ref101] Zhang Y. Mu Q. Zhou Z. Song H. Zhang Y. Wu F.. (2016a). Protective effect of irisin on atherosclerosis via suppressing oxidized low density lipoprotein induced vascular inflammation and endothelial dysfunction. PLoS One 11:e0158038. 10.1371/journal.pone.0158038, PMID: 27355581 PMC4927070

[ref102] Zhang Y. Song H. Zhang Y. Wu F. Mu Q. Jiang M.. (2016b). Irisin inhibits atherosclerosis by promoting endothelial proliferation through microRNA126-5p. J. Am. Heart Assoc. 5:e004031. 10.1161/JAHA.116.004031, PMID: 27671318 PMC5079047

[ref99] Zhang M. Xu Y. Jiang L. (2019). Irisin attenuates oxidized low-density lipoprotein impaired angiogenesis through AKT/mTOR/S6K1/Nrf2 pathway. J. Cell. Physiol. 234, 18951–18962. 10.1002/jcp.28535, PMID: 30942905

[ref98] Zhang H. J. Zhang X. F. Ma Z. M. Pan L. L. Chen Z. Han H. W.. (2013). Irisin is inversely associated with intrahepatic triglyceride contents in obese adults. J. Hepatol. 59, 557–562. 10.1016/j.jhep.2013.04.030, PMID: 23665283

[ref103] Zhu G. Wang J. Song M. Zhou F. Fu D. Ruan G.. (2016). Irisin increased the number and improved the function of endothelial progenitor cells in diabetes mellitus mice. J. Cardiovasc. Pharmacol. 68, 67–73. 10.1097/FJC.0000000000000386, PMID: 27002278 PMC4936438

